# From promoting to inhibiting: diverse roles of helicases in HIV-1 Replication

**DOI:** 10.1186/1742-4690-9-79

**Published:** 2012-09-28

**Authors:** Rene-Pierre Lorgeoux, Fei Guo, Chen Liang

**Affiliations:** 1 McGill AIDS Centre, Lady Davis Institute-Jewish General Hospital, Montreal, H3T 1E2, Quebec, Canada; 2 Department of Medicine, McGill University, Montreal, H3A 2B4, Quebec, Canada; 3 Department of Microbiology and Immunology, McGill University, Montreal, H3A 2B4, Quebec, Canada; 4 Institute of Pathogen Biology, Chinese Academy of Medical Sciences & Peking Union Medical College, Beijing, 100730, China

**Keywords:** HIV-1, helicase, RNP

## Abstract

Helicases hydrolyze nucleotide triphosphates (NTPs) and use the energy to modify the structures of nucleic acids. They are key players in every cellular process involving RNA or DNA. Human immunodeficiency virus type 1 (HIV-1) does not encode a helicase, thus it has to exploit cellular helicases in order to efficiently replicate its RNA genome. Indeed, several helicases have been found to specifically associate with HIV-1 and promote viral replication. However, studies have also revealed a couple of helicases that inhibit HIV-1 replication; these findings suggest that HIV-1 can either benefit from the function of cellular helicases or become curtailed by these enzymes. In this review, we focus on what is known about how a specific helicase associates with HIV-1 and how a distinct step of HIV-1 replication is affected. Despite many helicases having demonstrated roles in HIV-1 replication and dozens of other helicase candidates awaiting to be tested, a deeper appreciation of their involvement in the HIV-1 life cycle is hindered by our limited knowledge at the enzymatic and molecular levels regarding how helicases shape the conformation and structure of viral RNA-protein complexes and how these conformational changes are translated into functional outcomes in the context of viral replication.

## Review

Human immunodeficiency virus type 1 (HIV-1) is a lentivirus. Replication of the HIV-1 RNA genome involves reverse transcription by viral reverse transcriptase, integration into cellular DNA by viral integrase, and transcription by cellular RNA polymerase II. HIV-1 RNA is subject to the regulation by viral proteins including Tat, Rev, and Gag that recognize specific viral RNA structures. Tat binds to the TAR (transactivation response) RNA that is located at the very 5^′^ end of the viral genome, and it further recruits cellular factors including the P-TEFb (positive transcription elongation factor b) complex to the HIV-1 promoter and enhances transcription [1]. Rev recognizes the RRE (Rev response element) RNA that is located within the envelope protein-coding region, and it promotes the nuclear export of unspliced and partially spliced viral RNA via association with the CRM1 (chromosome region maintenance 1, also named exportin 1) nuclear export machinery [1]. Gag recognizes the viral RNA packaging signals located at the 5^′^ untranslated region (5^′^UTR) and recruits two copies of full-length HIV-1 RNA into each virus particle [2]. Recent studies have begun to reveal that a group of cellular proteins named helicases modulate HIV-1 replication through interacting with Tat, Rev and Gag proteins.

**Figure 1:**
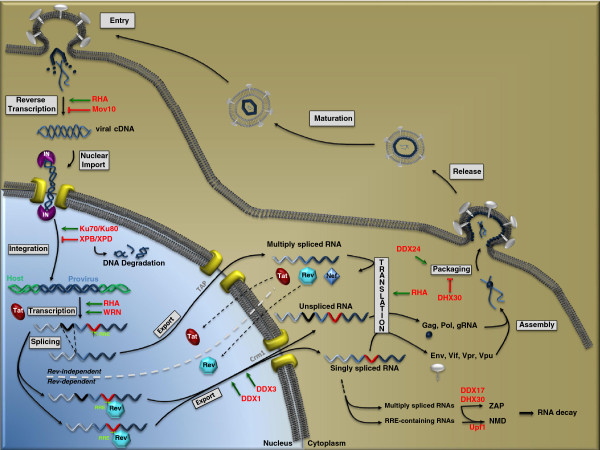
** Putative Roles of helicasesin HIV-1 life cycle.** HIV-1 infection starts with the entry step and ends with production of mature and infectious virus particles. The flow of the virus life cycle is narrated with arrows. Helicase involvement at distinct steps of HIV-1 life cycle is illustrated. Names of helicases are highlighted in red letters. Green arrows indicate a stimulating effect of a specific helicase on HIV-1 replication, the red lines denote inhibition.

The importance of helicases in HIV-1 replication began to receive much attention when DDX3 was identified as an essential factor of the Rev/CRM1/RRE RNA export complex in 2004 [3]. The role of helicases in HIV-1 replication was further highlighted in a 2006 review where the authors stated that the story of HIV-1 and helicase would continue to unfold [4]. Indeed, in the subsequent years, more helicases were discovered that not only promote but also, in some cases, restrict HIV-1 replication. This review is aimed at providing an up-to-date account of the HIV-1 and helicase story with a focus on helicases that exert specific association with Tat, Rev, Gag or viral RNA. We also briefly discuss the possible involvement of helicases in HIV-1 RNA packaging and viral DNA integration, as well as how HIV-1 evades recognition by the RNA helicase RIG-I. An overview is provided in Figure 1 to illustrate the helicases that are known to modulate distinct steps of HIV-1 replication. The general role of helicases in viral infection is also discussed in two recent excellent reviews [5,6]. Methods to study the activity of RNA helicases in the context of viral replication are described in [7]. 

## Helicases share conserved core structures and have diversified functions

Helicases are enzymes that hydrolyze NTPs (nucleotide triphosphates) and use the energy to unwind nucleic acid duplexes or to translocate along the nucleic acid strand [8]. They are ubiquitously expressed and participate in almost every cellular event involving nucleic acids. Helicases are characterized by two RecA-like domains that exhibit ATP-binding as well as nucleic acid-binding properties. Among the conserved motifs that helicases carry are the Walker A and B boxes that bind and hydrolyze NTPs, as well as the “arginine fingers” that couple NTP hydrolysis with nucleic acid unwinding or translocating activities. On the basis of their conserved motifs and enzymatic properties, helicases are classified into six superfamilies (SF) [8]. SFI and SFII have the most members, they function in a monomeric or a dimeric form. Many SF1 and SFII helicases contain the DExD/H motif [9]. Members of SFIII to SFVI are of viral or bacterial origin and often form hexamers [10,11]. Depending on whether they unwind RNA or DNA duplexes, helicases are also grouped as RNA helicases and DNA helicases. But this definition can become ambiguous for some helicases, such as RNA helicase A (RHA), that are able to unwind both RNA and DNA [12]. An RNA helicase database is now available at http://www.rnahelicase.org[13].

The biochemical properties of helicases can be defined by four measurable parameters. These include, 1) translocation rate, which represents the number of bases translocate per second; 2) directionality of action either from 5^′^ to 3^′^ or from 3^′^ to 5^′^; 3) processivity which is characterized by the number of rounds of catalysis before a helicase falls off the substrate; and 4) step size which represents the number of base pairs translocated during each NTP hydrolysis event [8]. Helicases differ considerably in their biochemical properties. For example, hexameric helicases often translocate long distance on DNA before falling off, whereas RNA helicases of the DEAD-box family unwind a short stretch of double-stranded RNA of no more than two helical turns. Even among DEAD-box helicases, they exhibit a wide array of activities [9]. For example, the DEAD-box protein eIF4AIII, upon binding to ATP, shows RNA clamping activity and serves to recruit the core components of exon junction complex (EJC) [14]. DDX21 (also named RH-II/GuA) acts as a strand annealer in an ATP-independent manner [15]. These biochemical properties often determine the biological functions of helicases.

Helicases are also regulated by co-factors. For example, the translation initiation factor eIF4A alone exhibits low ATP-dependent helicase activity [16]. Binding to eIF4B and eIF4H greatly enhances the ability of eIF4A to unwind RNA [17]. In addition, a local activation of the yeast helicase Dbp5 by inositol hexakisphosphate 6 (InsP6) and Gle1 leads to the removal of mRNA export factor Mex67 when mRNA arrives at the cytoplasmic side of the nuclear pore complex (NPC) [18,19]. Therefore, despite the conserved motifs and the similar folding that all helicase core domains share, each helicase is highly specific in its way to modify nucleic acid structures and regulate nucleic acid functions.

## Helicases as the co-factors of HIV-1 Tat

**Figure 2:**
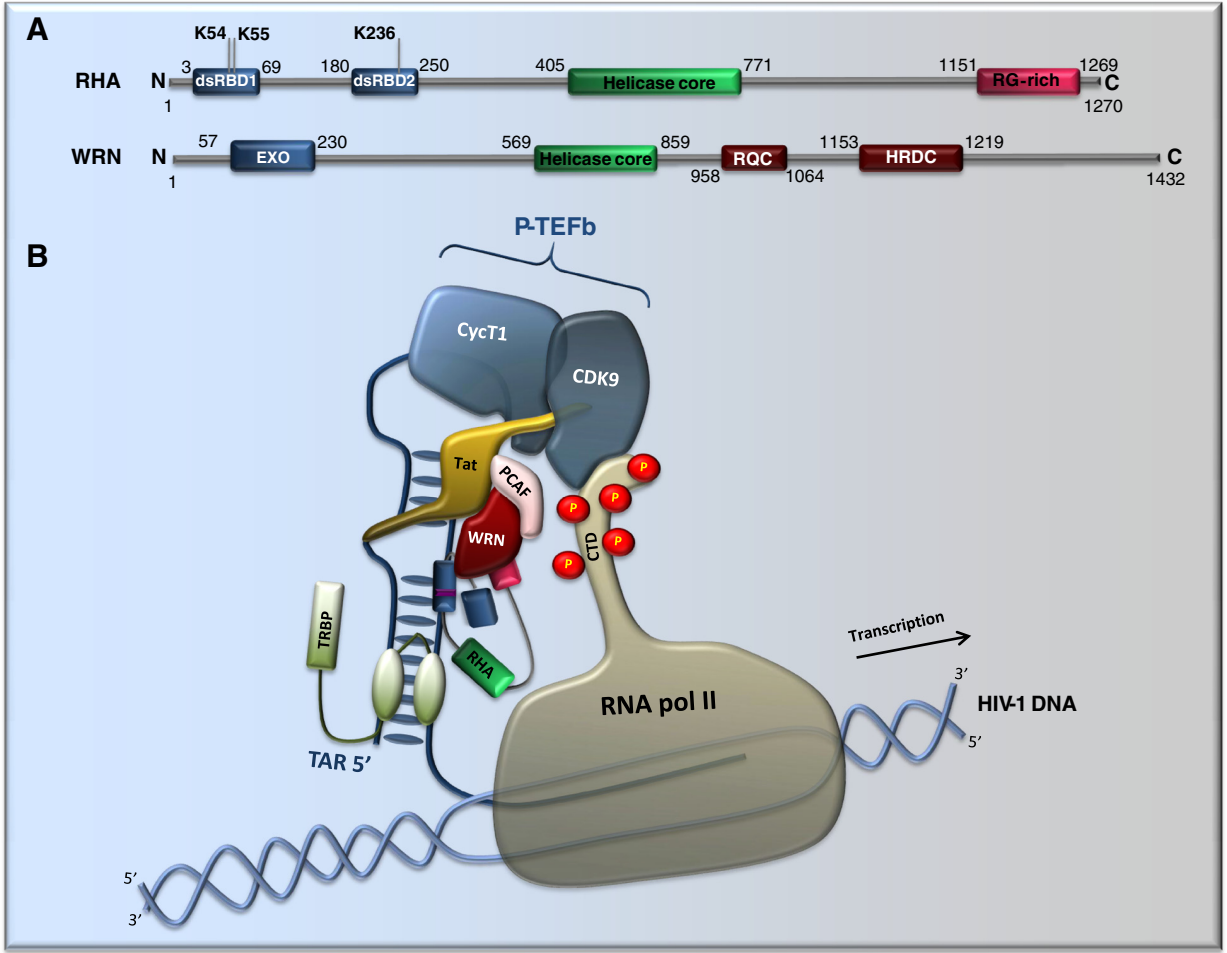
** Interaction of WRN andRHA with the TAR/Tatcomplex.** (**A**) Domain structures of RHA and WRN. The amino acid positions of each illustrated domain are indicated. dsRBD, double-stranded RNA-binding domain; RG-rich, arginine (R) and glycine (G)-rich region; EXO, exonuclease domain; RQC, RecQ Conserved domain; HRDC, helicase RNase D C-terminus domain. (**B**) WRN interacts with Tat and helps the recruitment of the P-TEFb complex (consisting of CycT1 and CDK9) to the HIV-1 promoter. RHA binds to the stem of TAR RNA via its dsRBD and also interacts with WRN. TRBP, TAR RNA binding protein.

HIV-1 Tat protein activates viral RNA synthesis [1]. Tat binds to the TAR RNA and recruits transcription factors to stimulate both transcription initiation and elongation. These transcription factors include p300/CREB-binding protein-associated factor (PCAF) and P-TEFb. P-TEFb contains cyclin T1 (CycT1) and cyclin-dependent kinase 9 (CDK9). CDK9 hyperphosphorylates the C-terminal domain (CTD) of RNA polymerase II and activates transcription elongation (Figure 2) [1]. In addition to these transcription factors, two helicases, the Werner syndrome (WRN) helicase and RHA, were reported to act as co-factors of Tat and enhance HIV-1 gene expression [20,21]. 

WRN is a member of the RECQ helicase family that also consists of RECQL (RECQ protein-like), BLM (bloom syndrome), RECQ4/RTS (Rothmund-Thomson syndrome) and RECQ5. RECQ helicases harbor the DEAH motif and belong to SFII. They are capable of resolving complex DNA structures that often block DNA replication fork progression [22]. In addition to its role in DNA recombination, WRN also promotes RNA polymerase II-dependent transcription, which is partially attributable to its ability to stimulate the DNA-unwinding activity of DNA topoisomerase I [23]. In line with its role in transcription, WRN was recently shown to interact with HIV-1 Tat and promote HIV-1 LTR transactivation (Figure 2) [20]. WRN and Tat are co-localized within the nuclei of HIV-1 infected cells. The purified recombinant GST-Tat is able to pull down the endogenous WRN. WRN appears to enhance HIV-1 gene expression by facilitating the recruitment of PCAF and P-TEFb to HIV-1 LTR [20]. In support of this role of WRN, ectopic expression of wild type WRN in human lymphocytes increases HIV-1 p24(Gag) production and viral replication.

In addition to the WRN helicase, RHA has also been shown to promote TAR-dependent HIV-1 gene expression [21]. RHA contains the DEIH Walker B motif, is a DEXH helicase. In addition to the helicase core domain, RHA has two double-stranded RNA-binding domains (dsRBDs) at its N-terminal region and the arginine- and glycine-rich (RGG) repeats at its C-terminal region (Figure 2) [24]. These latter domains target RHA to its RNA substrates. HIV-1 TAR RNA has been shown binding to the N-terminal dsRBDs of RHA [21,25]. This interaction allows RHA to affect a few steps of HIV-1 replication including transcription. Similar to WRN, RHA increases both basal activity from HIV-1 LTR and Tat transactivation (Figure 2) [21]. It is unclear whether RHA directly interacts with Tat as WRN does. Interestingly, the dsRBD II and the RGG repeats of RHA directly interact with the N-terminal exonuclease domain of WRN, and stimulate its exonuclease activity [26]. With such an interaction, RHA promotes the WRN-mediated degradation of D-loop DNA as well as the unwinding of Okazaki fragment-like hybrids [26,27]. It is thus conceivable that these two helicases may act together to promote HIV-1 RNA synthesis (Figure 2).

## The essential role of helicases in Rev-dependent RNA export

**Figure 3:**
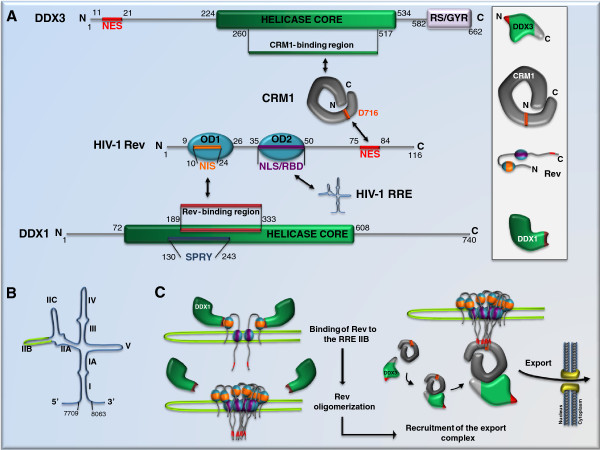
** DDX1 and DDX3 promoteRev-dependent RNA export.** (**A**) The domain structures of DDX3, Rev and DDX1 are illustrated. CRM1 binds to the helicase core of DDX3. DDX1 directly interacts with Rev. The protein regions involved in these interactions are highlighted. NES, nuclear export signal; NLS, nuclear localization signal; OD1, oligomerization domain 1; RBD, RNA binding domain. (**B**) The secondary structures of the RRE RNA. The IIB stem-loop is highlighted in light green which serves as the binding site of Rev. (**C**) A hypothetical model to illustrate the roles of DDX1 and DDX3 in Rev-mediated RNA export. DDX1 binds to Rev and promotes Rev oligomerization on the RRE IIB. Then the oligomerized Rev molecules recruit the CRM1/DDX3 binary complex (one or multiple copies) and together export viral RNA into the cytoplasm.

The intron-containing cellular RNA cannot leave the nucleus before they are completely spliced. HIV-1 needs to evade this form of cellular surveillance in order to export its full-length and partially spliced RNA into the cytoplasm and produce viral structural proteins and accessory proteins. This viral evasion relies on the Rev protein that binds to the HIV-1 RNA sequence RRE and communicates the intron-containing HIV-1 RNA to the CRM1 nuclear export pathway for export [28]. Crossing the NPC (nuclear pore complex) is not a trivial task for the RNP (ribonuclear protein) complex. Remodeling is required so that the RNP is able to thread through the NPC channel. Snay-Hodge and colleagues first reported in 1998 that in yeast, the Dbp5 RNA helicase (human DDX19 homolog) associates with the NPC and is essential for mRNA export [29]. It was later shown that following activation by Gle1 and InsP6 at the cytoplasmic face of the NPC, Dbp5 remodels mRNP and removes RNA transporters Mex67 (human TAP homolog) and Mtr2 (human NXT1 (nuclear transport factor 2-like export factor 1) homolog) [18,19] (Table 1). It remained unknown whether a similar role of helicase is required for Rev/CRM1/RRE-mediated export of intron-containing HIV-1 RNA until Yedavalli and colleagues reported the essential role of DDX3 in this export event (Figure 3) [3]. It is noted that, in addition to helicases, other cellular factors have also been shown to promote Rev/RRE-mediated RNA export. One such example is Mtr3 that binds to the Rev/RRE complex and facilitates the export of HIV-1 RNA [30,31]. 

**Table 1:** **Human genes vs Yeasthomologs**

**Human Name**	**Yeast homolog**
DDX19	Dbp5
TAP, NXF1	Mex67
GLE1	Gle1
IP6K	InsP6
NXT1	Mtr2
SKIV2L2	Mtr4
PAPD7	Trf4

DDX3 is a DEAD-box protein (Figure 3A). Although DDX3 has been shown to interact with RNA transport factors TAP/NXF1 and REF/Aly, it does not appear to play a role in bulk mRNA export [32-34]. It is interesting to note that Ded1 (yeast DDX3 homolog) modulates translation by controlling the conformation of eIF4F-mRNA complex [35], suggesting a role of Ded1/DDX3 in translation. The function of DDX3 in RNA export was not recognized until DDX3 was found to participate in the Rev-dependent export of unspliced and partially spliced HIV-1 RNAs [3]. Rev is co-immunoprecipitated with DDX3, but a direct interaction between the two proteins has not been experimentally demonstrated. Rather, the purified GST-CRM1 is able to pull down the *in vitro* translated DDX3. This direct interaction depends on the DDX3 fragment at amino acid positions 260 to 517 that does not include the NES (nuclear export signal) sequence, and is Ran-GTP independent (Figure 3A, 3C), which suggests that instead of a cargo, DDX3 acts as an effector in the CRM1-mediated nuclear export pathway.

In support of the important role of DDX3 in Rev-dependent HIV-1 RNA export, knockdown of DDX3 or expression of the dominant negative mutant of DDX3 significantly diminishes HIV-1 replication [36]. Mutation of a unique fragment between the helicase motifs I and Ia diminishes the ability of DDX3 to bind to HIV-1 RNA and impairs HIV-1 replication [37]. Interestingly, a ligand of this unique region reduces HIV-1 infection of HeLaP4 cells, suggesting the possibility of targeting this domain to abrogate the function of DDX3 in HIV-1 replication. It remains to be tested whether DDX3 is involved in CRM1-mediated export of cellular RNAs such as snRNA and rRNA, and to elucidate the molecular details regarding how DDX3 promotes RNA export.

In addition to DDX3, the RNA helicase DDX1 has also been reported to associate with Rev and promote the export of RRE-containing viral RNA (Figure 3A) [38]. Purified DDX1 exhibits RNA-dependent ATPase activity. The DDX1 sequence from amino acids 189 to 333 directly interacts with the nuclear inhibitory signal (NIS) at amino acids 10 to 24 in Rev. Through this interaction, DDX1 promotes Rev oligomerization on the RRE RNA (Figure 3B, 3C) [39,40]. This function of DDX1 is important because coordinated binding of multiple copies of Rev, rather than Rev monomer, to the RRE is required for initiating RNA export [41]. In support of its role as a co-factor of Rev, the low DDX1 level in astrocytes results in a predominant cytoplasmic location of Rev, which partially accounts for the poor susceptibility of this cell type to HIV-1 infection [42]. On the basis of these observations, we propose that DDX1 and DDX3 act sequentially in the Rev-dependent RNA export (Figure 3C). DDX1 first binds to Rev and promotes Rev oligomerization on the RRE RNA. Then the oligomerized Rev molecules, through presenting multiple copies of NES, recruit the CRM1/DDX3 complex that subsequently exports the RRE-containing HIV-1 RNA into the cytoplasm.

A recent proteomic study led to the finding of more helicases that associate with HIV-1 Rev [43]. In addition to DDX1 and DDX3, these include DDX5, DDX17, DDX21, DHX36, DDX47 and RHA. Silencing DDX5, DDX17 or DDX21 significantly modulates the production of HIV-1 particles, suggesting a functional role of these helicases in HIV-1 replication. It remains to be further investigated how each of these helicases affects the function of Rev and whether they play redundant roles or are involved in distinct steps of Rev-mediated RNA export. Interestingly, these helicases were not reported to associate with Rev in a separate protoemic study that employed the affinity tagging purification and mass spectrometry methods to identify cellular factors that interact with each of the 18 HIV-1 proteins [44]. This discrepancy may reflect the RNA-dependent nature of the Rev-helicase interaction.

## Helicases in HIV-1 particles

Gag makes HIV-1 particles [45]. In addition to viral RNA and viral proteins, a variety of cellular factors find their way into virus particles via direct or indirect interactions with Gag [46,47]. Among the many cellular factors are two helicases, RHA and MOV10 (Moloney leukemia virus 10 homolog) [48-51]. These two helicases both affect HIV-1 reverse transcription but with opposite outcomes.

RHA interacts with Gag in an RNA-dependent manner [48]. Knockdown of RHA in virus producer cells diminishes the infectivity of progeny HIV-1 particles, suggesting a functional role of the presence of RHA in the virions [25,48]. This deficit in infectivity is caused at least in part by decreased viral reverse transcription [25,48]. A further analysis of the viral RNA complex within the RHA-depleted virus particles reveals a reduced level of tRNALys.3 that is annealed onto the primer binding site (PBS) [52]. This latter finding is verified by *in vitro* study showing that the purified recombinant wild type RHA, but not its helicase-null mutant K417R, assists Gag/NC in promoting the formation of tRNALys.3/viral RNA binary complex [52]. Moreover, this binary viral RNA complex that is formed with the assistance of RHA exhibits higher efficiency in reverse transcription [52], which suggests that RHA not only promotes the annealing of tRNALys.3 onto viral RNA but also helps the viral RNA complex to adopt conformations in favor of the action of viral reverse transcriptase.

**Figure 4:**
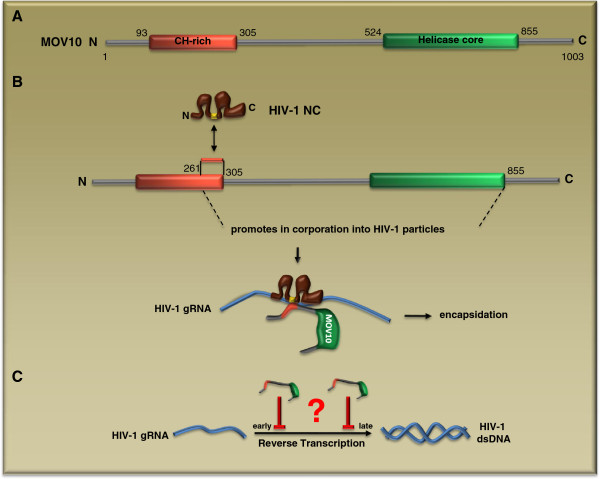
** MOV10 inhibits HIV-1 reversetranscription.** (**A**) Domain structures of MOV10. MOV10 carries a CH-rich (cysteine/histidine-rich) domain in the N-terminal region and a helicase core domain in the C-terminal region. (**B**) The basic linker of NC (highlighted in yellow) interacts with the (aa 261-305) region of MOV10, which contributes to the incorporation of MOV10 into HIV-1 particles. (**C**) MOV10 diminishes HIV-1 reverse transcription either at the early or the late phase of the reaction.

**Figure 5:**
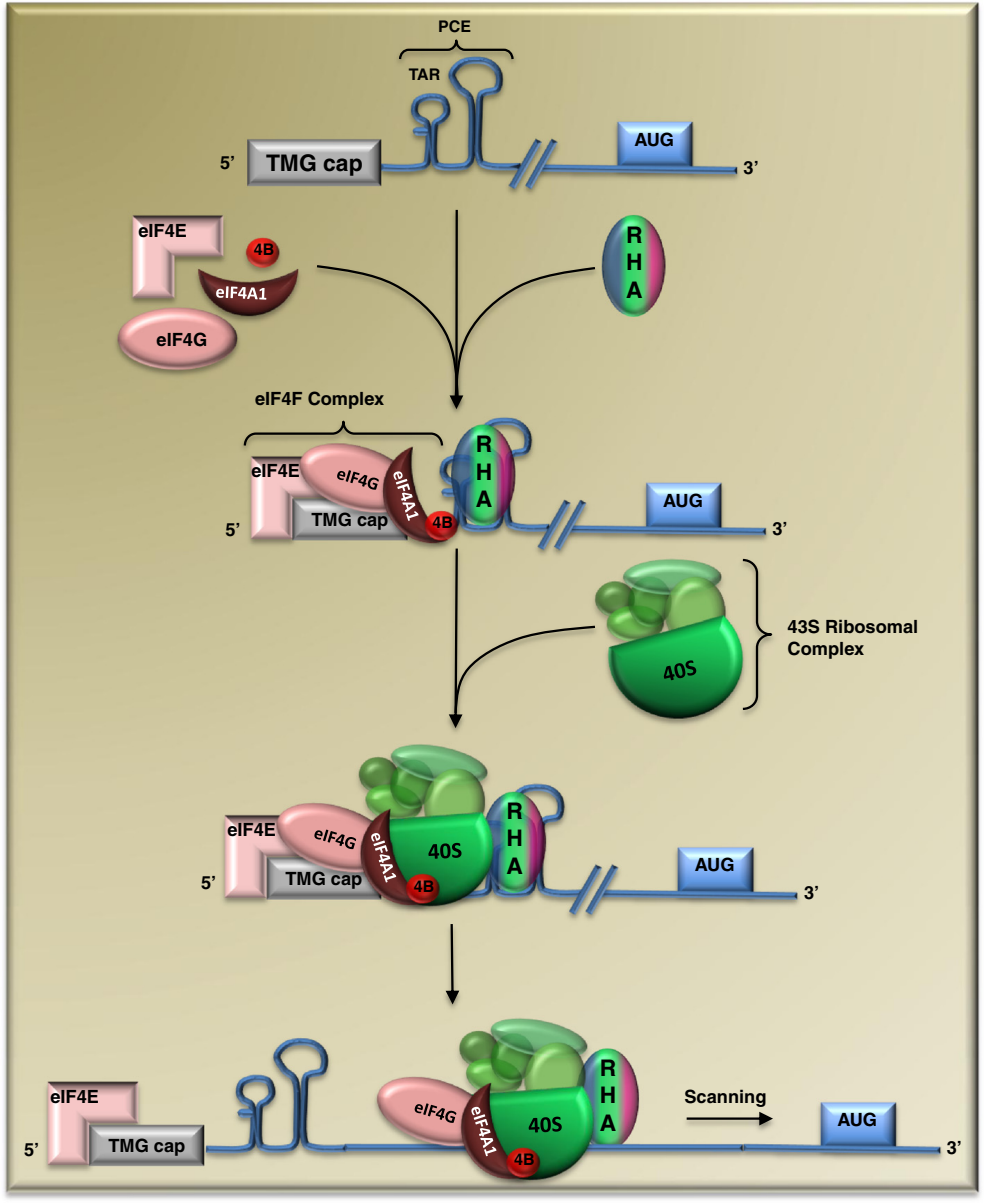
** RHA stimulates HIV-1 proteintranslation.** Translation initiation factor eIF4E binds to the 5^′^cap structure of HIV-1 RNA together with eIF4G and eIF4A1 in the context of the eIF4F translation initiation complex, which further recruits the 43S ribosomal complex to the mRNA. RHA binds to the R/U5 region of HIV-1 RNA and is proposed to assist the 43S ribosomal complex to scan the HIV-1 5^′^UTR and locate the translation start codon AUG.

In contrast with the stimulatory role of RHA in HIV-1 reverse transcription, MOV10 exerts an inhibitory effect [49-51]. MOV10 is a SFI RNA helicase, and has the DEAG Walker B motif. In addition to the helicase core domain, MOV10 has a long N-terminal region that bears a cysteine- and histidine-rich (CH) domain (Figure 4A). Detailed mutagenesis analysis showed that the MOV10 sequence at amino acid positions 261 to 305 interacts with the basic linker of the NC domain of Gag protein (Figure 4B) [53]. In addition, efficient viral incorporation of MOV10 also requires the helicase core domain downstream of this (261 to 305) region (Figure 4B). It remains controversial in terms of which step of viral reverse transcription is suppressed by MOV10. Burdick and colleagues reported a defect at the late stage of reverse transcription [51], whereas Wang et al and Furtak et al observed a reduction in the yield of early HIV-1 cDNA products (Figure 5C) [49,50]. Testing purified MOV10 in cell-free HIV-1 reverse transcription assays is one way to elucidate the molecular details of its inhibition activity. The ability of MOV10 to dampen reverse transcription may enable it to have a role in controlling endogenous retroelements. Indeed, two recent studies reported that MOV10 inhibits the retrotransposition of both LTR and non-LTR endogenous retroelements including LINE-1, Alu and IAP [54,55]. 

MOV10 may not act alone to inhibit HIV-1 reverse transcription. It is known that MOV10 binds to Ago2 and is a player in the microRNA pathway [56]. This function allows MOV10 to control local protein translation at synapses and modulate synaptic plasticity [57]. Ago2 was recently detected in HIV-1 particles [58]. This latter finding raises the possibility that MOV10 and Ago2 may be packaged into virus particles as one complex and, together, modulate the function of viral RNA.

## RHA enhances HIV-1 RNA translation

The role of RHA in HIV-1 replication goes beyond enhancing viral transcription and reverse transcription. RHA also increases HIV-1 RNA translation (Figure 5) [25]. This function of RHA depends on its binding to the R/U5 sequence of HIV-1 RNA that has been named the post-transcriptional control element (PCE). The PCE exists in the 5^′^UTRs of different retroviruses, including spleen necrosis virus (SNV), Mason-Pfizer monkey virus (MPMV), human foamy virus (HFV), reticuloendotheliosis virus strain A (REV-A), human T-cell leukemia virus type 1 (HTLV-1), feline leukemia virus (FeLV), and bovine leukemia virus (BLV) [59]. Studies show that RHA augments translation by promoting the association of PCE-containing RNA with polyribosomes [60]. This translation mechanism may have a cellular origin, since the translation of cellular junD mRNA is stimulated by RHA in a 5^′^UTR-dependent manner [61].

RHA is not the only helicase that promotes the translation of mRNA having structured 5^′^UTR. DHX29 has been reported to facilitate the formation of the 48S translation initiation complex on the AUG codon of mRNAs such as neutrophil cytosolic factor 2 (NCF2) and CDC25 that harbor secondary structures at their 5^′^UTRs [62,63]. These findings suggest that in addition to the RNA helicase eIF4A that functions as a conical translation initiation factor, the translation of specific mRNA may benefit from the action of other helicases [17].

RHA contributes to one of the several translation mechanisms that HIV-1 has harnessed to ensure efficient production of viral proteins. First, the activity of internal ribosome entry site (IRES) has been detected in the HIV-1 5^′^UTR and the Gag-coding region [64,65], which allows translation to initiate in a cap-independent fashion. It has been noted that HIV-1 PCE and IRES are mapped to different sequences of the 5^′^UTR [25,65], indicating that they represent distinct translation mechanisms. Second, the Rev/RRE-exported viral RNAs have a trimethylguanosine (TMG) cap at their 5^′^ ends as opposed to the 7-methylguanosine (m7G) at the 5^′^ end of most cellular mRNA [66]. The TMG cap is synthesized by the PIMT enzyme (peroxisome proliferator-activated receptor-interacting protein with methyltransferases) that is recruited to HIV-1 RNA through binding to Rev. As a result, PIMT increases the translation of Rev-exported viral RNA. It is postulated that this mechanism ensures the production of optimal amounts of viral structural proteins at the late stage of HIV infection to produce virus particles. This finding also explains why Rev, besides its role in RNA export, also enhances translation [67,68].

These different translation mechanisms contribute to HIV-1 protein production at different levels and under different conditions. RHA, through binding to R/U5 that is present on both spliced and unspliced HIV-1 RNAs, promotes the synthesis of all HIV-1 proteins, whereas the TMG cap, whose formation is Rev-dependent, increases the translation of viral structural proteins. In regard to HIV-1 IRES, its activity is cell cycle-dependent and responds to oxidative stress [65,69,70].

## Upf1 associates with the 3′UTR of HIV-1 RNA

**Figure 6:**
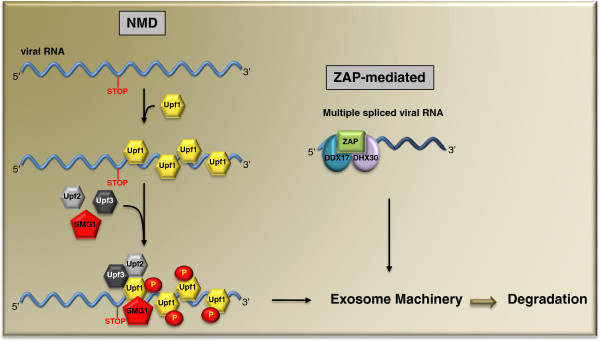
** Roles of Upf1 andZAP in HIV-1 RNAdegradation.** Multiple copies of Upf1 first bind to the long 3^′^UTR and then recruit Upf2, Upf3 and SMG1 to assemble the NMD machinery. The exosomes are then recruited to degrade RNA. ZAP, together with DDX17 and DHX30, recognizes multiply spliced HIV-1 RNA and sends the RNA to exosomes for degradation.

HIV-1 RNA has a long 3^′^UTR that represents one of the signals, in addition to the pre-mature termination codon (PTC) and the upstream open reading frame (uORF), that are recognized by the nonsense-mediated decay (NMD) machinery [71]. This long 3^′^UTR scenario is particularly true for the unspliced HIV-1 RNA in which the termination codon of the *gagpol* gene is located approximately 4 kb from the 3^′^ end of viral RNA. Although much is known about PTC-triggered NMD, it remained unclear how sensing the length of 3^′^UTR is achieved by the NMD machinery until Hogg and Goff reported the association of Upf1 with HIV-1 3^′^UTR in an RNA length-dependent manner (Figure 6) [72]. 

Upf1 is a key component of the NMD core machinery [71]. As an SFI RNA helicase, Upf1 exhibits nucleic acid-dependent ATPase activity and 5^′^ to 3^′^ RNA unwinding activity [73]. Using the RNA affinity purification technique and the mass spectrometry method, Hogg and Goff discovered that Upf1 associates with HIV-1 3^′^UTR and other model 3^′^UTRs [72]. When the abundance of Upf1 association with a 3^′^UTR exceeds a certain threshold, the RNA is marked as a potential substrate for NMD. Frequent translation readthrough counters this mechanism by displacing Upf1 from the 3^′^UTR [72]. Interestingly, rare translation readthrough also rescues the RNA from NMD without affecting the association of Upf1 with 3^′^UTR, which suggests a two-step model for Upf1 to sense 3^′^UTR and to potentiate decay. Since HIV-1 and other retroviruses use the frame shift mechanism to read through the stop codon of Gag in order to produce the Gag-Pol polyprotein, this translation mechanism may protect HIV-1 RNA from Upf1-mediated RNA decay. In support of this possible counter measure, it has been reported that Rous sarcoma virus (RSV) has the RSV stability element (RSE) that contains a frame shift pseudoknot and prevents the Upf1-dependent degradation of unspliced RSV RNA [74]. However, the story of Upf1 and HIV-1 may be more complicated. One study shows that the knockdown of Upf1 in HeLa cells leads to decreased levels of both HIV-1 RNA and viral Gag protein and that this observation is independent of the role of Upf1 in NMD [75]. This study concluded that HIV-1 has evolved to use Upf1 to stabilize viral RNA. Further studies are warranted to define how Upf1 modulates HIV-1 replication in HIV-1 natural target cells such as primary CD4+ T cells.

In addition to Upf1, HIV-1 RNA was recently shown being subject to ZAP (zinc finger antiviral protein)-mediated degradation (Figure 6) [76]. ZAP was originally reported to inhibit murine leukemia virus (MLV) infection [77]. Interestingly, ZAP causes the degradation of multiply spliced HIV-1 RNA while sparing the unspliced and singly spliced viral RNA [78,79]. This degradation process can be initiated either by shortening the 3^′^ polyadenylation tail or by removing the 5^′^ cap. Two RNA helicases, DDX17 and DHX30, were found as co-factors of ZAP [76,80,81], which may function by remodeling the viral RNP and assisting the recruitment RNA degradation machinery. A similar role of RNA helicase in cellular RNA degradation has been reported for Mtr4 in yeast that bridges the TRAMP (Trf4/Air2/Mtr4 polyadenylation) complex to exosomes and remodels substrate RNA molecules [82,83].

## DDX24 and DHX30 modulate HIV-1 RNA packaging

Each HIV-1 particle packages two copies of unspliced viral RNA that are non-covalently linked via the RNA stem-loop structure SL1 that is defined as the dimerization initiation site (DIS) [2,84,85]. The NC domain of Gag is primarily responsible for recognizing the RNA packaging signals that comprise the SL1, SL2 and SL3 RNA structures at the 5^′^UTR [86,87]. The nuclear magnetic resonance (NMR) structures of the 712-nt HIV-1 5^′^-leader RNA reveal a structure-based coordination of HIV-1 RNA packaging, dimerization and translation [88]. In addition to these cis-acting viral RNA signals at the 5^′^UTR, the Rev/RRE system has also been shown to significantly augment HIV-1 RNA packaging [89]. A direct involvement of helicases in HIV-1 RNA packaging has not been documented, although it is known that bacteriophages use helicases as motors to “thread” phage DNA into their capsids [90]. Nonetheless, a couple of helicases have been implicated in modulating the genome packaging of some retroviruses including HIV-1. For example, DDX6 was reported to affect the viral genome packaging of foamy virus, a spumaretrovirus [91]. Relocation of DDX6 from P bodies and stress granules to virus assembly sites at the perinuclear region was seen in cells infected with foamy virus. However, no interaction was detected between DDX6 and Gag, and DDX6 was not seen in the virus particles [91]. As opposed to the reported role of DDX6 in foamy virus assembly, DDX6 binds to HIV-1 Gag and promotes Gag assembly, independent of viral RNA packaging [92]. We previously observed that knockdown of the RNA helicase DDX24 diminishes HIV-1 RNA packaging [93]. This effect was seen only for Rev/RRE-exported, not for CTE (constitutive transport element)-exported viral RNA, which likely results from the interaction of DDX24 with Rev. With its predominant location within the nucleolus, DDX24 may gain access to HIV-1 RNA through association with Rev and participates in viral RNA remodeling. The effect may then extend to the viral RNA packaging event that takes place within the cytoplasm. In contrast to the stimulatory effect of DDX24, another RNA helicase, DHX30, inhibits HIV-1 RNA packaging [94], which may be attributable to its accessory role in ZAP-mediated HIV-1 RNA degradation [80].

## The putative role of helicases in HIV-1 DNA integration

Integration of HIV-1 DNA into cellular DNA is catalyzed by viral integrase in the context of pre-integration complex (PIC) [95,96]. The PIC consists of the full-length HIV-1 DNA, integrase, viral and cellular factors that assist viral DNA integration. In addition to a number of cellular proteins such as BAF (barrier-to-autointegration factor), Gemin2, EED (embryonic ectoderm development), integrase interactor 1, and LEDGF/p75 (lens epithelium-derived growth factor), the helicase DDX19A was recently shown to likely associate with the PIC [97]. Using the yeast two-hybrid method, Studamire and Goff screened for cellular proteins that interact with the integrase of Moloney murine leukemia virus (MoMLV) [98]. The candidates include several helicases such as Ku70/XRCC6, DDX5 and DDX18. It would be interesting to test whether these helicases also associate with HIV-1 integrase and whether they play a functional role in HIV-1 DNA integration. It should be noted that no helicase has ever been shown experimentally to interact with HIV-1 integrase, therefore a direct role of helicase in HIV-1 DNA integration remains to be established.

Despite not being components of the PIC, helicases in the DNA repair machinery may participate in HIV-1 DNA integration in an indirect manner. For example, unintegrated HIV-1 DNA has been reported to be the substrate of the non-homologous DNA end joining (NHEJ) pathway [99]. Knockdown of the Ku80 DNA helicase, a key player in NHEJ, reduces HIV-1 DNA integration and diminishes viral replication in human CEM4fx cells [99]. In one study, 232 host DNA repair proteins were silenced using siRNA oligos and the effects on HIV-1 DNA integration were measured [100]. The targeted proteins are involved in base excision repair (BER), nucleotide excision repair (NER), NHEJ, single strand break repair (SSBR), double strand break repair (DSB), mismatch repair (MMR), and homologous recombination (HR). The results revealed an important role of the BER pathway in HIV-1 DNA integration [100,101]. Notably, knockdown of a few DNA repair helicases including ERCC3 and RECQL4 diminishes HIV-1 infection, suggesting their role in viral DNA integration [100].

Instead of assisting HIV-1 DNA integration, certain DNA repair machineries exert inhibitory effects. For example, NER-deficient cells that are mutated in the helicases XPB and XPD are more susceptible to transduction by HIV-based retroviral vectors owing to an increase in the integrated viral DNA [102,103]. This suggests a role of these two DNA helicases, and likely via the underlying NER pathway, in defending cells against retroviral integration. In conclusion, no helicase has been reported to specifically interact with HIV-1 integrase and thereby modulate directly viral DNA integration. Evidence does suggest that some helicases become involved in HIV-1 DNA integration in the context of DNA repair pathways.

## RIG-I is curtailed by viral protease for sensing HIV-1 RNA

A small family of DExD/H helicases including RIG-I (retinoic acid-inducible gene I), MDA-5 (melanoma differentiation associated protein-5) and LGP2 (laboratory of genetics and physiology 2) recognize viral RNA and trigger interferon production [104]. Although transfecting the monomeric and dimeric HIV-1 RNA into cells triggers interferon production in a RIG-I-dependent manner, HIV-1 infection of monocyte-derived macrophages does not induce interferon response [105]. This suggests that HIV-1 has a mechanism to evade the recognition by RIG-I. Indeed, further studies showed that HIV-1 protease removes RIG-I from the cytosol to an insoluble fraction, therefore inhibiting RIG-I-mediated antiviral signaling [105].

## Conclusions

HIV-1 engages helicases to facilitate viral replication at different steps. This engagement is achieved by interacting with helicases via either viral RNA or viral proteins. For example, RHA binds to the R/U5 region of HIV-1 RNA and promotes viral gene expression and viral reverse transcription [21,25,48,52]. DDX1 and DDX3 are associated with the Rev/RRE/CRM1 complex and regulate viral RNA export [3,38,40]. Also, WRN interacts with Tat and elevates HIV-1 gene expression [20]. Recruiting these helicases to viral RNP complexes at different stages of viral replication reflects the need of HIV-1 to harness cellular helicases to overcome certain rate-limiting steps of viral RNA replication or to accomplish an activity that rarely occurs to cellular RNA such as reverse transcription. Opening the door to cellular helicases also exposes the virus to helicases that are deleterious to HIV-1 replication. One such example is MOV10 that finds its way into HIV-1 particles and impairs viral reverse transcription [49-51].

More cellular helicases than described herein may associate with HIV-1, given that a dozen of helicases have been reported in several genome-wide functional screens that were aimed at identifying cellular proteins that modulate HIV-1 infection. These include DDX10, DDX19, DDX33, DDX53, DDX50, DDX55, DDX60L, FBXO18, IGHMBP2, YTHDC2, HFM1, RECQL4, RUVBL2 [97,106-108]. Furthermore, studies have also shown that HIV-1 infection alters the expression of a handful of cellular helicases [109,110]. Last but not least, in a recent study aimed at comprehensively mapping the interactions between cellular factors and each of the HIV-1 18 proteins, DDX49 was reported to associate with Gag, DDX20 with Vpr, and RECQ1 with Pol [44]. An important future task is to characterize the interactions of these candidate helicases with HIV-1 and to decipher their functions in HIV-1 infection.

How many helicases does HIV-1 really need? How many of these helicases play redundant roles in HIV-1 replication? In addition to these questions, we also lack a detailed knowledge at the molecular and enzymatic levels regarding how a helicase promotes or impedes a specific step of HIV-1 replication. It will be a challenging task to determine experimentally when and where a specific helicase becomes associated with HIV-1 RNPs, and it is also challenging to discern the structural and biochemical changes that a specific helicase can introduce into HIV-1 RNPs. Knowing these biochemical and enzymatic details will not only help to further elucidate the role of a helicase in HIV-1 RNA metabolism, but will also aid in the discovery of helicase inhibitors that may have the potential for treating HIV-1 infection [111].

## Competing interests

The authors declare that they have no competing interests.

## Authors’ contributions

RL, FG and CL wrote different sections of this manuscript. RL prepared the figures and the table. All three authors read and approved the manuscript.
